# [Corrigendum] CD271^+^ stem cell treatment of patients with chronic stroke: A retrospective case series report

**DOI:** 10.3892/etm.2023.12243

**Published:** 2023-10-06

**Authors:** Felician Stancioiu, Georgios Z. Papadakis, George Lazopoulos, Demetrios A. Spandidos, Aristidis Tsatsakis, Marius Floroiu, Corin Badiu

Exp Ther Med 20:2055–2062, 2020; DOI: 10.3892/etm.2020.8948

Subsequently to the publication of the above paper, an interested reader expressed concerns as to the nature of the paper (what category of article the paper should have been assigned to), whether important information may have been missing from the article, and whether or not the work should have formed the basis a clinical trial.

In response, the authors have explained that this article was not conceived as a clinical study, but as a case series report, resulting from non-standardized administration of treatments to individual patients on a case-by-case basis (under the EU rules governing compassionate care and hospital exemption), not under homogenous inclusion/exclusion criteria applicable to a clinical study. Moreover, the ensuing patient data were analysed retrospectively, and the intent of the article was to provide insights into this type of treatment, for which further developments and improvements are still needed.

In consequence, the following changes are proposed for this article (the changed text is highlighted in **bold**):

i) as shown above, the title of the article has been changed to ‘CD271^+^ stem cell treatment of patients with chronic stroke: **A retrospective case series report**’, to reflect that this study should be regarded as a case series report;

ii) it should have been explicitly stated in the text that the patient data were analysed retrospectively, and that the intent of the article was to provide some preliminary insights into this type of treatment, which is still in need of further developments and improvements;

and iii) on p. 2061, the left-hand column, the first sentence in the final paragraph of the Discussion would have read better as follows: ‘**Based on above data and the cited literature on mesenchymal stem cells - of which CD271^+^ represent a subpopulation - it can be said that** treating chronic stroke patients with CD271^+^ stem cells administered intrathecally and/or intravenously is a safe therapeutic option with good results especially for spasticity and aphasia; at the same time it needs to be further studied and improved...’.

These oversights did not have an impact on the conclusions reported in this study, and all the authors agree to the publication of this Corrigendum.

